# A Multicenter prospective study of poor-grade aneurysmal subarachnoid hemorrhage (AMPAS): observational registry study

**DOI:** 10.1186/1471-2377-14-86

**Published:** 2014-04-17

**Authors:** Bing Zhao, Xianxi Tan, Hua Yang, Kuang Zheng, Zequn Li, Ye Xiong, Ming Zhong

**Affiliations:** 1 Department of Neurosurgery, The first affiliated hospital of Wenzhou Medical University, Wenzhou, China; 2 Department of Neurosurgery, The affiliated hospital of Guiyang Medical University, Guiyang, China

**Keywords:** Aneurysmal subarachnoid hemorrhage, Poor-grade, Definitive management, Outcome, Predicators

## Abstract

**Background:**

Poor-grade aneurysmal subarachnoid hemorrhage (aSAH) is associated with very high mortality and morbidity. Our limited knowledge on predictors of long-term outcome in poor-grade patients with aSAH definitively managed comes from retrospective and prospective studies of small case series of patients in single center. The purpose of the AMPAS is to determine the long-term outcomes in poor-grade patients with different managements within different time after aSAH, and identify the independent predictors of the outcome that help guide the decision on definitive management.

**Methods/design:**

The AMPAS study is a prospective, multicenter, observational registry of consecutive hospitalized patients with poor grade aSAH (WFNS grade IV and V). The aim is to enroll at least 226 poor-grade patients in 11 high-volume medical centers (eg, >150 aSAH cases per year) affiliated to different universities in China. This study will describe poor grade patients and aneurysm characteristics, treatment strategies (modality and time of definitive management), hospitalization complications and outcomes evolve over time. The definitive management is ruptured aneurysm treatment. Outcomes at 3, 6, 12 months after the management were measured using the Glasgow Outcome Scale and the Modified Rankin Scale.

**Discussion:**

The AMPAS is the first prospective, multicenter, observational registry of poor grade aSAH with any management. This study will contribute to a better understanding of significant predictors of outcome in poor grade patients and help guide future treatment of the worst patients after aSAH.

**Trial registration:**

Chinese Clinical Trial Registry: ChiCTR-TNRC-10001041.

## Background

Aneurysmal subarachnoid hemorrhage (aSAH) is a significant health problem with a mortality rate of about 60% within 6 months [1]. Although poor-grade aSAH accounts for 20-30% of patients with aSAH [2], the overall good outcome with surgical or endovascular treatment is only 33-56%, and mortality rate is 28–58% [3-5]. Poor-grade aneurysm patients received no surgical treatment with 75-100% mortality [6,7]. Unlike good grade aSAH, poor grade patients often have an acute brain injury caused by severe cerebral swelling associated with intracerebral hemorrhage [8], acute hydrocephalus or intraventricular hemorrhage [5], microcirculatory disturbances or decreased cerebral perfusion [9], and increased intracranial pressure [10]. Therefore, the clinical presentation of poor grade aSAH is highly complex ranging from comatose patients with or without midbrain symptoms [11] to neurogenic cardiac and pulmonary dysfunction [9] or final multi-organ failure. These differences in presentation also represent different severity of brain injury and acute complications associated with aSAH. Therefore, it is possible that careful selected patients with poor grade could achieve good outcome after a patient-specific treatment [7,12-14].

However, selection of patients for management on the basis of the neurological condition remains controversial [15-17]. There are many different treatment modalities in poor grade patients: aneurysm surgical treatment, endovascular treatment, aggressive management of acute hydrocephalus or ventricular hemorrhage without aneurysm treatment, and conservative medication. Early treatment (within 72 hours after aSAH) of patients with a good grade on admission (WFNS I-III) prevents the rebleeding of aneurysm and leads to a significantly better outcome [1]. However, recent studies have not identified a superior treatment option for patients with poor grade aSAH. Although some retrospective studies also demonstrated carefully selected patients with early aneurysm treatment had a good outcome, treatment strategies for poor grade aSAH are difficult to make between the family and the treating physician owing to the high mortality and morbidity poor-grade aSAH [18,19]. There have been two randomized treatment clinical trials comparing endovascular and surgical treatment of aSAH, particularly of good grade patients [20,21]. These results suggested a potentially better effect of endovascular treatment than surgical treatment. However, there was a low recruitment rate of poor-grade patients., and ISAT included mostly good grade aSAH patients with small anterior circulation aneurysms [22]. Nowadays the largest data on outcome of patients with poor-grade aSAH was only a retrospective case series of 283 patients [23]. But the retrospective study only determined the outcome at discharge after surgical obliteration of the aneurysm. Whether poor grade patients will benefit from advanced endovascular techniques and materials is still unknown, therefore, which is a better treatment strategy for poor grade aSAH still remains controversial.

In addition, many selection bias and different condition grading and results of poor outcome described in many studies exists in the literatures. Different definitions of poor grade aSAH included Hunt& Huns grade IV and V [4,6], WFNS grade IV and V [12,19,24]. The grading scale was also measured either on admission or before aneurysm treatment. Moreover, outcomes were measured using the Glasgow outcome score (GOS) [13,16] or the modified Rankin Scale (mRS) [25,26] at different time of follow-up, and the good outcome was also identified differently [22,27], for example, good outcome was identified as the mRs ≤2 or the mRS ≤3. These data in small numbers of selected patients treated with definitive treatments in a single center were collected for a retrospective study. Also, most patients treated over the past several years or ten years in were also included in these studies. There could be selection bias among retrospective studies. Therefore, we could not make a good multivariable analysis.

With advancements in early surgical treatment, endovascular treatment, multimodality neuromonitoring and multidisciplinary management, we need a better understanding long-term outcome in poor grade patients within different treatment options within different time after aSAH. Therefore, we performed the prospective multicenter study on predictors of outcome in poor grade aSAH, and we could further embark a suitable randomized trial on the basis of registry data.

## Methods/design

### Study design

The AMPAS is a prospective, multicenter, non-randomized, observational registry study of consecutive case series. Eleven high-volume medical centers (eg, >150 aSAH cases per year) that offers endovascular and microsurgical treatment of aneurysm and neurological intensive care services were included in the study. These academic centers are large hospitals of different medical universities in China.

### Ethical approval and governmental funding

The final study protocol and written informed consent were approved by Chinese Ethics Committee of Registering Clinical Trials (ChiECRCT-2010019). This study was funded by the Chinese Ministry of Health (WKJ2010-2-016), Wenzhou Bureau of Science and Technology (Y20090005). All informed consent was obtained from the patient legal representative.

### Objectives

The key purpose of the AMPAS study is to determine the long-term outcomes in poor grade patients with different managements within different time after aSAH, and identify the independent predictors of the outcome. That will help guide the decision on definitive management of poor grade patients and the design of a future randomized clinical trial.

This study will also determine the following questions: (1) which clinical variables before and after any management are associated with long-term outcome? Medical history, clinical condition on admission, condition before and after aneurysm treatment, aSAH CT grading scale, aneurysm location and size, modality and time of definitive management, and complications during the hospitalization. (2) What are the most common complications related to different managements (endovascular, microsurgical treatment, and surgical management without aneurysm repair or intensive monitoring and medication)?

### Study population

#### Patients

All consecutive patients were diagnosed with SAH confirmed by head CT scans or lumbar puncture in the emergency department. The patient condition was first evaluated by neurosurgeons using the WFNS grade. If the patient with WFNS grade IV and V was hospitalized to be eligible for entry in the registry. But the following inclusion and exclusion criteria were also applied.

#### Inclusion criteria

(1) age ranging from 18 to 75 years old.

(2) aneurysm confirmed by computed tomography angiography (CTA) or digital subtraction angiography (DSA).

(3) poor-grade patients with WFNS grade IV or V on admission or before aneurysm treatment.

(4) ruptured aneurysm correlated with the current SAH;

(5) Informed consent.

#### Exclusion criteria

(1) withdrawing any managements in the emergency department.

(2) SAH resulting from other reasons and unclear diagnosis of aneurysm.

(3) aneurysm treated with endovascular or surgical treatment at any other referring hospitals.

(4) pregnant or lactating women.

(5) loss of breath.

(6) patients with severe systemic disorders and expected life span less than one year.

### Detailed clinical data

Clinical variables included medical history such as hypertension, smoking and diabetes, clinical condition after the initial aSAH, on admission, before treatment, neurological condition evaluated by the GCS, Hunt&Hess, WFNS grade, herniation, ruptured aneurysm location and size, timing of definitive management, treatment procedure recording, neurological condition within 72 hours after the treatment, complications during the hospitalization, follow-up imaging and the presumed reasons of death. The total of hospital expense was also recorded.

### Management protocol

All eligible poor-grade patients were managed in neurological intensive care unit (NICU) according to the guidelines for the management of aSAH [28,29]. The management protocol included aggressive resuscitation such as intubation and ventilation, microsurgical treatment, endovascular treatment, surgical control intracranial pressure using external ventricular drainage, and neurological intensive care. Therefore, treatment modalities were divided into two patterns: definitive management of ruptured aneurysm; aggressive management including only surgical treatment of hydrocephalus [5,25], ventricular hemorrhage or intracranial hypertension, and intensive monitoring and medication. Time of definitive management was also grouped: ultra-early management (within 24 hours after aSAH), early management (between 24 hours and 72 hours, late management (more than 72 hours). Aneurysm treatment was discussed with cerebrovascular surgeons and endovascular specialists based on the patient age and aneurysm characteristics and location. Treatment options were discussed between the family and the treating physician. The final decision was made by the family.

In general, surgical treatment of aneurysm was preferred for patients with aSAH associated with large intracranial hematoma (>30 ml) and clinical signs of brainstem compression. The treatment included aneurysm clipping or wrapping, hematoma evacuation, or decompressive craniectomy. Endovascular treatment of aneurysm included aneurysm coiling, balloon or stent assisted coiling, or a parent artery occluded. External ventricular drainage was inserted in patients with acute hydrocephalus or ventricular hemorrhage before or after aneurysm obliteration. All patients received intravenous nimodipine, Mannitol and hypervolemic, hypertensive, hemodilution (3H) therapy after definitive management. Patients remained in NICU until medically stable for transfer or until the family terminated the treatment.

### Follow-up and data quality

All patients will be followed up after the management by a neurosurgeon using the telephone interview or in-person interview. The neurosurgeon was trained before the AMPAS registry and was not involved in the treatment of poor-grade patients. Outcomes at 3, 6, and 12 months was measured using the GOS and mRS. The mRS of 0–2 was identified as good outcome [22], and the score of 3–6 is generally as poor outcome.

All data were collected using a written case report form (CRF) and an electronic case report form (eCRF) through a registry website using a center-specific login and password. Meanwhile, data verification was undertaken in 20% of all cases to assess the accuracy of data recording. If the written CRF did not match eCRF, the case record was excluded. If there were more than 5 patients excluded in the primary verification and more than 5 patients without available clinical variables and follow-up in the registry, All of patients in the center were totally excluded at the time of database closure.

### Sample size and data analysis

No data is available about the rate of poor outcome in unselected population of poor grade patients. According to previous literature, poor grade account for 20% of aSAH [2], and poor come in selected patients after surgical or endovascular treatment is about 55%. The target number of patients included in the registry is at least 226 to identify the proposed 20 predictors of the outcome with a two-sided significance level of 5%, a power of 80% and an anticipated effect size of 0.10. The primary expected number of patients is more than 252 in case there are about 10% of patients lost to follow-up. Data was presented as mean and standard deviation for continuous variables or frequency for categorical variables. Significances between variables were analyzed using the t-test or Chi-square test. Association between clinical variables and outcome will be analyzed, and predictors of long-term outcome were identified using a univariate and multivariate analysis. The difference was expressed as an odds ratio (OR, with 95% confidence interval [CI]), and significance was considered if P value was <0.05.

## Discussion

The long-term outcomes in poor grade patients within different treatment options within different time after aSAH should be determined with advancements in definitive management and multidisciplinary monitoring and intensive care [30]. This study described poor-grade patients with aSAH and aneurysm characteristics, treatment strategies (modality and time of definitive management), and hospitalization complications, and outcomes evolve over time. The prospective multicenter study on predictors of outcome in poor grade aSAH will be performed, and a suitable randomized trial will be embarked on the basis of registry data.

Predictors of poor outcome in poor-grade patients have been demonstrated in several retrospective case series studies [3,16,23,31] or few prospective case series [26,32] over the decades. However, these results of predictors remain controversial with the small numbers of patients in a single center [32] or selection bias such as collecting patient data in different eras [26] or careful selection of poor grade patients [13]. Only one prospective, multicenter trial of 184 poor grade patients in a study of the calcium antagonist nimodipine in 1988 showed that factors prognostic for outcome were surgical treatment, neurological grade on admission, age, initial systolic blood pressure, and aneurysm size [18]. Another study using prospectively maintained SAH database in single center between 1996 and 2002, only included 40% of the 98 definitively treated patients. This study demonstrated that significant predictors of poor outcome were patient age older than 65 years, hyperglycemia, worst preoperative Hunt and Hess Grade V, and aneurysm size of at least 13 mm. Also, a controlled observational study of 51 consecutive patients treated with endovascular coiling within 96 hours of aSAH indicated persistent intracranial pressure elevation and higher mean 8-day S100B value independently predicted the 1-year outcome [32]. In addition, there was only one retrospective review of large number of case series (283 cases) at multiple centers in Japan [23]. But the study only identified the independent predictors of outcome at discharge including advanced age, WFNS grade V, improvement in WFNS grade, and low-density area associated with vasospasm on CT, whereas rebleeding, early aneurysm surgery and treatment modality (surgical clipping or coil embolization) were not independently associated with outcome in poor-grade Patients [23].

Although, there is one ongoing prospective, single-center, observer-blinded, randomized controlled trial to determine optimal timing for surgery in poor-grade patients [33], and one pragmatic, multicenter, randomized trial comparing clinical outcomes for patients with aSAH allocated to coiling or clipping [22], evidence for the predictors of long-term outcome in poor grade patients has not been provided so far. Moreover, a recent systematic review also showed clinical prediction models for aSAH used a few simple predictors and have not had external validation for clinical or research purposes [34]. Therefore, and further study will be validated reliable prediction models for poor grade aSAH.

In conclusion, the AMPAS study will be the first prospective, multicenter, observational registry of poor grade aSAH with any management worldwide, particularly in China. This study will contribute to a better understanding of significant predictors of outcome in poor grade patients and help guide future treatment of the worst patients after aSAH.

### Current status

Between October 2010 and March 2012, 366 poor-grade aSAH patients have been entered. The last patient follow-up at 12 months was completed until March 2013. Data of 76 patients in 2 centers were totally excluded at the time of database closure according to data quality policy because clinical variables and follow-up were lost. At last, Of 293 poor grade patients on admission or before aneurysm treatment in the registry, 168 patients with WFNS grade IV, and 125 patients with WFNS grade V were included.234 patients (80%) were definitively treated with surgical treatment(103patients) and endovascular treatment (131 patients). 20 patients only received aggressive management including external ventricular drainage, and hematoma evacuation and decompressive craniectomy.39 patients only received neuromonitoring and medical treatment.

## Appendix

AMPAS Investigators

Hongqi Zhang, Beijing Xuanwu Hospital affiliated to Capital Medical university; Chuangsheng liang, China Medical University;Huaizhang Shi, Harbin Medical University;Jin XU, Zhejiang Medical University;Li Pan, Wuhan General Hospital of Military Medical University;Xin Zhang, Nanjing General Hospital of Military Medical University; Gang Zhu, West South hospital of the Third Military Medical University; Jianping Deng,Tangdu Hospital of the Fourth Military Medical University.

## Abbreviations

aSAH: aneurysmal subarachnoid hemorrhage; AMPAS: A multicenter prospective study of poor-grade aneurysmal SAH; ISAT: WFNS: World federation of neurological surgeons grading scale; NICU: Neurological intensive care unit; EVD: External ventricular drainages; CTA: Tomography angiography; DSA: Digital subtraction angiography; GCS: Glasgow coma scale; GOS: Glasgow outcome scale; mRS: modified rankin score; CRF: Case report form (CRF); eCRF: Electronic case report form.

## Competing interests

The authors declare that they have no competing interests.

## Author’s contributions

MZ obtained research funding and supervised the registry study. BZ, XT and MZ developed this study protocol. BZ, XT, HY, KZ, ZL, XY, MZ and AMPAS investigators all participated in the final design of the study. BZ drafted the first manuscript. All authors read and approved the final manuscript.

## Pre-publication history

The pre-publication history for this paper can be accessed here:

http://www.biomedcentral.com/1471-2377/14/86/prepub
